# Case Report: Neonatal Multi-System Inflammatory Syndrome Associated With SARS-CoV-2 Exposure in Two Cases From Saudi Arabia

**DOI:** 10.3389/fped.2021.652857

**Published:** 2021-05-13

**Authors:** Lana A. Shaiba, Adnan Hadid, Khalid A. Altirkawi, Hind M. Bakheet, Aminah Mohammed Alherz, Shaik Asfaq Hussain, Badr H. Sobaih, Abdulrahman M. Alnemri, Rana Almaghrabi, Medina Ahmed, Maria A. Arafah, Abdullah Jarallah, Elham Essa Bukhari, Fahad A. Alzamil

**Affiliations:** ^1^Department of Pediatrics, College of Medicine, King Saud University, Riyadh, Saudi Arabia; ^2^Neonatology, King Saud University Medical City, Riyadh, Saudi Arabia; ^3^Pediatric Infectious Disease, King Saud University Medical City, Riyadh, Saudi Arabia; ^4^Department of Pediatrics, Qatif Central Hospital, Qatif, Saudi Arabia; ^5^Pediatric Infectious Disease, Pediatric Department, Prince Sultan Military Medical City, Riyadh, Saudi Arabia; ^6^Microbiologist, Prince Sultan Military Medical City, Riyadh, Saudi Arabia; ^7^Department of Pathology, King Saud University, Riyadh, Saudi Arabia; ^8^Pathology, King Saud University Medical City, Riyadh, Saudi Arabia; ^9^Department of Cardiac Sciences, College of Medicine, King Saud University, Riyadh, Saudi Arabia; ^10^Pediatric Cardiology, Cardiac Science, King Saud University Medical City, Riyadh, Saudi Arabia

**Keywords:** neonatal, multi-system, inflammatory syndrome, SARS-CoV-2, vertical transmission

## Abstract

**Background:** Vertical transmission of SARS-CoV-2 is under investigation. A few reports suggest the possibility of SARS-CoV-2 transmission from mothers to their neonates. Most neonates have mild symptoms, but some develop multisystem involvement and shock.

**Case Presentation:** We report two cases of possible SARS-CoV-2 vertical transmission from mothers to their neonates. The first case shows maternal infection with SARS-CoV-2 in the second trimester followed by recurrent infection in the third trimester right before the delivery. The infant demonstrated respiratory distress soon after delivery along with myocardial dysfunction and multi-organ system involvement. The second case shows maternal infection with SARS-COV-2 at the time of delivery with preterm labor secondary to placental abruption, with that delivery resulting in the preterm neonate requiring non-invasive ventilation with multisystem involvement in the context of persistently positive SARS-COV-2 PCR in the neonate. Both neonates were treated with IVIG along with steroids. Both neonates recovered fully and were discharged and allowed to go home.

**Conclusion:** In neonates, COVID-19 usually presents as an asymptomatic or mild illness; some may develop a more severe course. Our two cases, however, demonstrate that multisystem involvement, although rare, is possible. This report also supports the current evidence of possible vertical transmission from mothers to their neonates. This multisystem involvement might be underreported and should be considered in neonates with respiratory distress when born to mothers suffering of COVID-19.

**Clinical Trial Registration:** [KSUMC], identifier [No#98763298].

## Introduction

Severe acute respiratory syndrome coronavirus 2 (SARS-CoV-2) infection during pregnancy may increase the risk of stillbirth, neonatal death, preterm birth, low birth weight, fetal distress, and neonatal asphyxia (1). The possibility of perinatal vertical transmission of SARS-CoV-2 seems to be very rare (2–4). A large prospective national cohort study in the UK included 66 neonatal confirmed SARS-CoV-2 infections; 17 infants were delivered to mothers known to have perinatal SARS-CoV-2 infection, and only two of them were vertically infected (5). The low placental expression of canonical receptors, with negligible co-transcription of angiotensin converting enzyme (ACE2) and transmembrane protease serine 2 (TMPRSS2) in the placenta necessary for the virus entry, may explain this low risk for vertical transmission (6). The SARS-CoV-2 virus was reported as one of the rare causes of fetal inflammatory response syndrome (FIRS) (7) and is associated with multisystem inflammatory syndrome in children (MIS-C) (8). Here we present two case series of two premature newborn infants with multisystem involvement and possible vertical transmission, in which one had high specific immunoglobulin for SARS-CoV-2, whereas the other had persistent positive nasopharyngeal swabs for SARS-CoV-2 virus since the age of 24 h.

## Case Presentation

### Case 1

A 36-week gestational age female infant, weighing 3,004 g, was delivered *via* induced vaginal delivery to a 33-year-old Filipino primigravida lady, at King Saud University Medical City (KSUMC), Riyadh, KSA. The mother works as a staff nurse at the same hospital. The mother tested positive for the SARS-CoV-2 virus in the second trimester after exposure to a COVID-19 patient, during which she was asymptomatic, and the repeated swab after 14 days was negative. But 19 days prior to delivery, she tested positive again for SARS-CoV-2 after exposure to a COVID-19 patient, when she developed mild upper respiratory symptoms which were managed at home. Repeated nasopharyngeal swabs remained positive until the 10th day after delivery (29 days from her first positive swab). The mother underwent induction of labor at 36 weeks of gestation due to preeclampsia.

**Figure 1:**
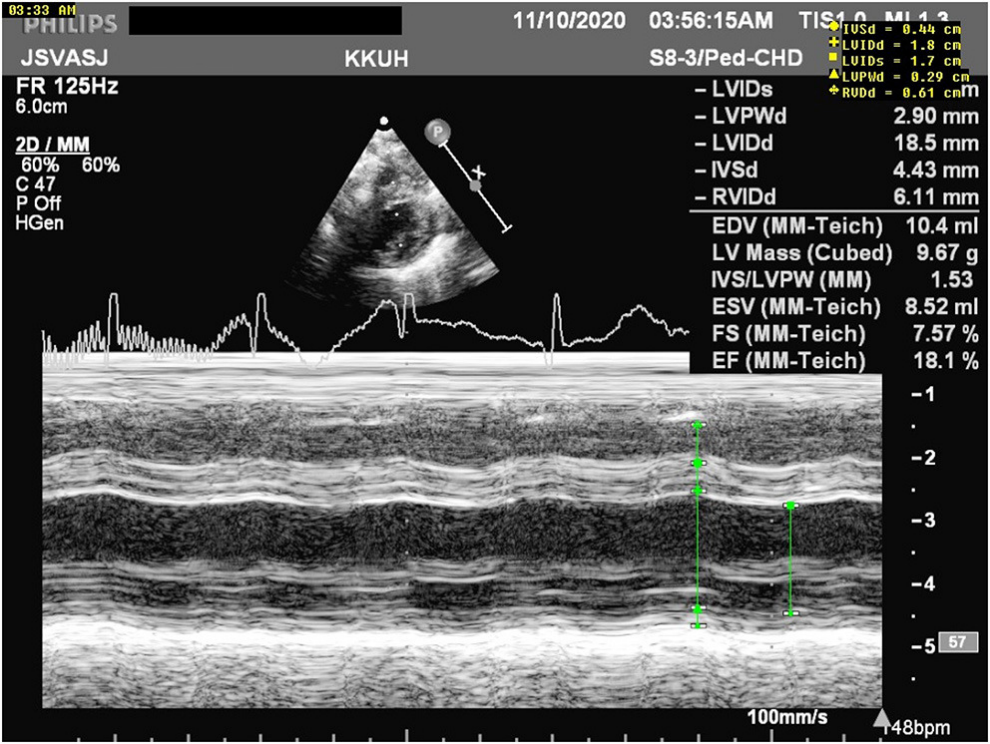
M mode measurements revealing poor cardiac function with an ejection fraction (EF) of 18.1%.

**Figure 2:**
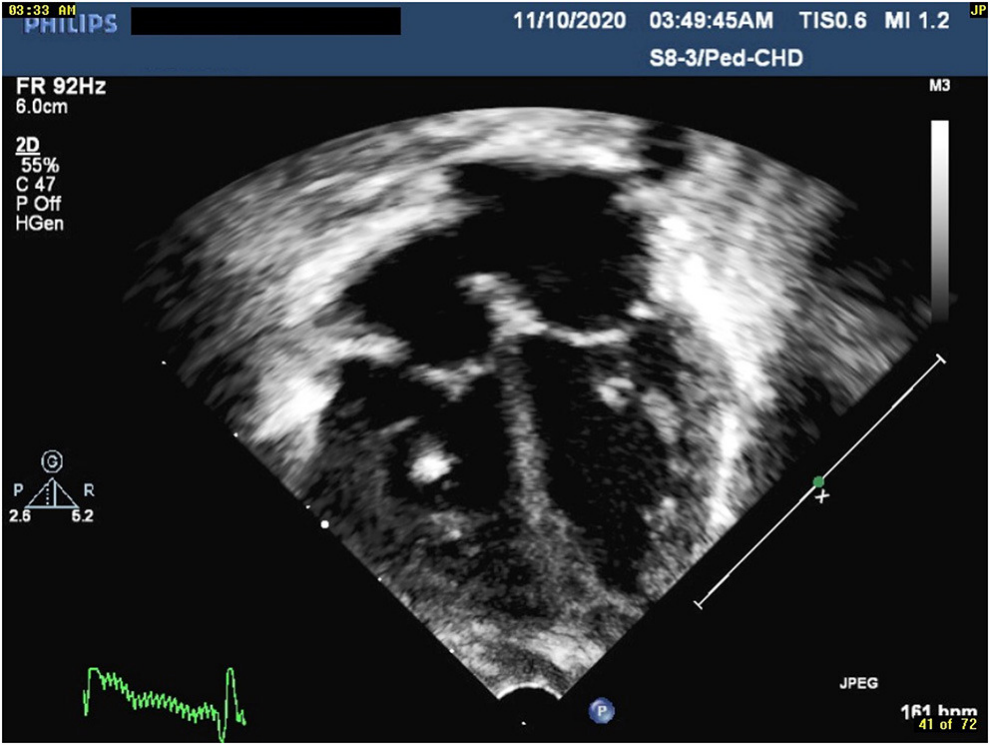
Four chambers view revealing shining chordae tendineae indicating ischemic changes.

Immediately after delivery, the baby was separated from the mother and admitted to an isolated room. The baby required no resuscitation, with Apgar scores of 8 and 9 at 1 and 5 min, respectively. At 15 min of life, she developed mild cyanosis with progressive respiratory distress. The chest radiograph was within normal limits, and she was supported by continuous positive airway pressure (CPAP), but oxygen saturation (SpO_2_) values remained low (70%) on FiO_2_ of 100%; thus, she was admitted to an isolation room in the NICU, where she was intubated and provided with conventional mechanical ventilation. Nonetheless, oxygenation remained low, so we shifted the respiratory support to high frequency oscillator ventilation (HFOV). An urgent echocardiograph was requested and prostaglandin E1 (PGE1) was initiated, as a duct-dependent cardiac lesion was suspected, with minimal improvement (Figures 1, 2). The echocardiograph at 2 h of age showed normal cardiac anatomy, a moderately dilated left ventricle (LV) with poor systolic function, echogenic papillary muscles (could be secondary to ischemia vs. acidosis), and widely patent *ductus arteriosus* (PDA) with a bidirectional shunt. The impression was a case of myocarditis for which PGE1 was discontinued, and dobutamine was administered for 48 h to manage left ventricular dysfunction. Given persistent metabolic acidosis and the bidirectional PDA, suggesting possible pulmonary hypertension, inhaled Nitric Oxide (iNO) was started, resulting in a significant improvement. A repeated echograph on the 2nd day of life showed improved LV ejection fraction to 68% and a closed *ductus arteriosus*.

Initial arterial blood gas showed severe metabolic acidosis (Table 1) with high lactate concentration (13.8 mmol/L). A nasopharyngeal sample for SARS-CoV-2 PCR at 9 h of life was negative. The blood investigations showed lymphopenia, thrombocytopenia, and low hemoglobin (Hb). Antibiotics (ampicillin and gentamycin) were administered after drawing blood cultures and continued for 7 days.

**Table 1:** Laboratory characteristics of case 1.

**General hematology**	**DOL: 1**	**DOL: 2**	**DOL: 3**	**DOL: 4**	**DOL: 5**	**DOL: 6**	**DOL: 7**	**DOL: 8**	**DOL: 12**
WBC	9.8	15.7	9.9	5.3	6.3	7.7		7.8	13.4
Hgb	119 (L)	139 (L)	133 (L)	130 (L)	128 (L)	114 (L)		122 (L)	126 (L)
Hct	45.3 (L)	39.6 (L)	38.0 (L)	36.8 (L)	35.3 (L)	32.5 (L)		43.3 (L)	35.7 (L)
Platelets	149	178	143	116 (L)	98 (L)	93 (L)		160	395
Neutro Auto#	2.8 (L)	12.1	4.3 (L)	1.6 (L)	4.7 (L)	3.0 (L)		2.0	4.9
Neutro Auto %	29.1 (L)	76.8 (H)	44.1 (L)	30.6(L)	75 (H)	38.3 (L)		25.5 (L)	36.2
Lympho Auto#	6.5	2.3	4	3.4	1.3 (L)	4.2		4.6	7.3
Lympho Auto %	66.1 (H)	14.5 (L)	40.3 (H)	63.3 (H)	21 (L)	54.8 (H)		59.8 (H)	54.2 (H)
ESR	QNS		QNS		9				
PT	34.2 (H)	19.8 (H)							
INR	2.59 (H)	1.47 (H)							
APTT	32.90	44.30 (H)							
Fibrinogin					3.91				
ALT	119 (H)	148 (H)	209 (H)	205 (H)	159 (H)	129 (H)	98 (H)		
AST	713 (H)	524 (H)	491 (H)	281 (H)	116 (H)	65 (H)	42 (H)		
Ammonia	89								
BUN	4.4	4.7	3.9	2.8	1.7	2.3	2.5	1.7	3.2
Creatinine Lvl	71	85	48	26 (L)	26 (l)	28	24	25	33
LDH	2,696 (H)		2.387 (H)						568 (H)
GGT	378 (H)	222 (H)	169 (H)	131 (H)	118 (h)	98 (h)	117 (H)		
Ferrtin					384.4 (h)				
Lactic Acid	> 15								
Uric Acid			257						
BNP			3,433 (H)						
Total CK	4,273 (H)		2,774 (H)						
Troponin-I			130 (H)						138.1 (H)
Procalcitonin	73.07 (H)			1.22 (H)					
CRP		15		9.57					
SARS-COV-2 IgG	4.30 (H)								
SARS-COV-2 IgG Interp	Positive								
COVID-19	Negative		Negative	Negative	Negative	Negative	Negative	Negative	Negative

The coagulation profile showed prolonged INR, for which she was given fresh frozen plasma (FFP), and liver enzymes were elevated suggesting liver injury. Cardiac enzymes (creatinine kinase = 4,273 unit/L and BNP = 3,433 pg/ml) were elevated, so was LDH (2,696 unit/L). Inflammatory markers were high (procalcitonin = 73.07 ng/ml and CRP = 15 mg/L; see Table 1), despite the negative blood cultures. iNO and inotropes (dobutamine) were discontinued on the third d of life, and the baby was shifted to conventional mechanical ventilation 1 d later.

On the 5th d of life, the diagnosis of multisystem inflammatory syndrome in children (MIS-C) was made, based on the multisystem involvement and the presence of highly specific immunoglobulins for SARS-CoV-2. Two doses of IVIG (1 g/kg/dose), and a course of IV hydrocortisone (0.5 mg/kg every 12 h for 7 d and then 0.5 mg/kg every 24 h for 3 d) were administered. On the 6th d of life, the baby was extubated successfully. The previously abnormal laboratory results (decreased platelets and lymphocytes counts, elevated liver enzymes, LDH, CRP, and procalcitonin levels) all revealed significant improvements (Table 1). In the first case study, we estimated levels of IgG only in the newborn baby, and they were found positive. We did not evaluate the mother's IgG and IgM levels.

**Figure 3:**
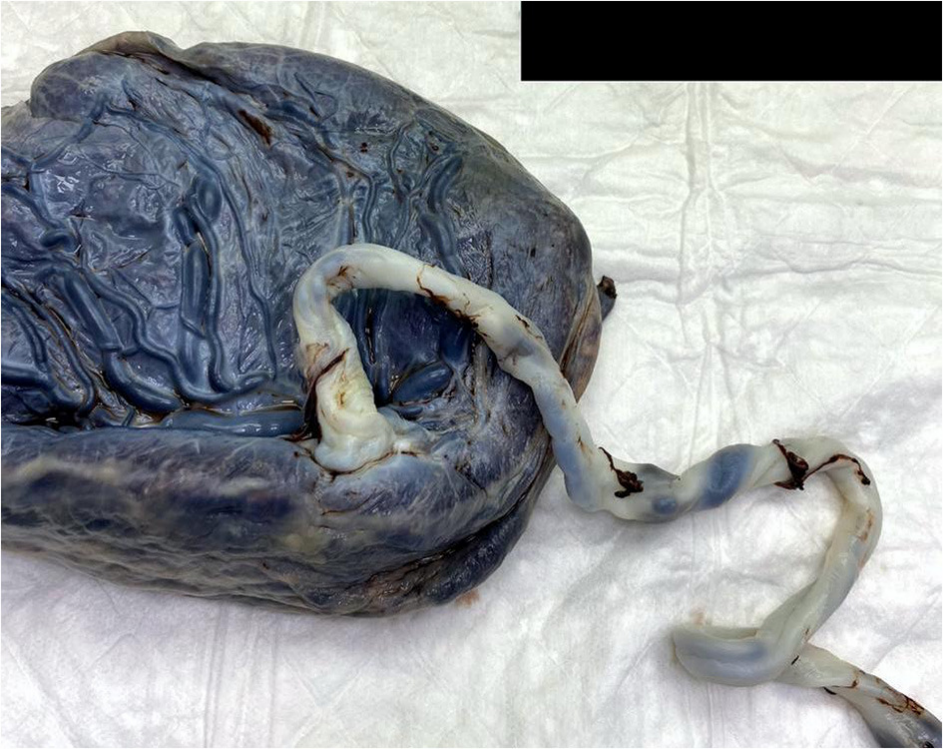
A macroscopic image of a singleton placenta with a peripherally inserted umbilical cord showing a reduced coiling index.

**Figure 4:**
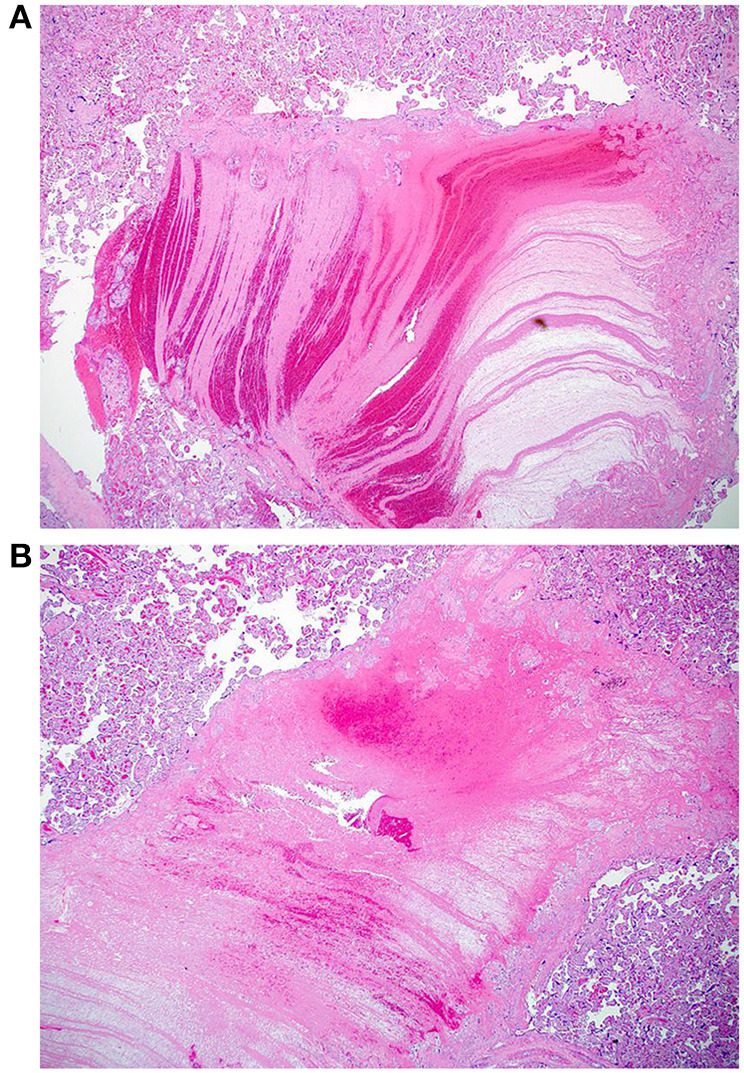
**(A,B)** Photomicrographs of two intervillous hematomas seen in case 1 (Hematoxylin and eosin stain, magnification x20).

**Figure 5:**
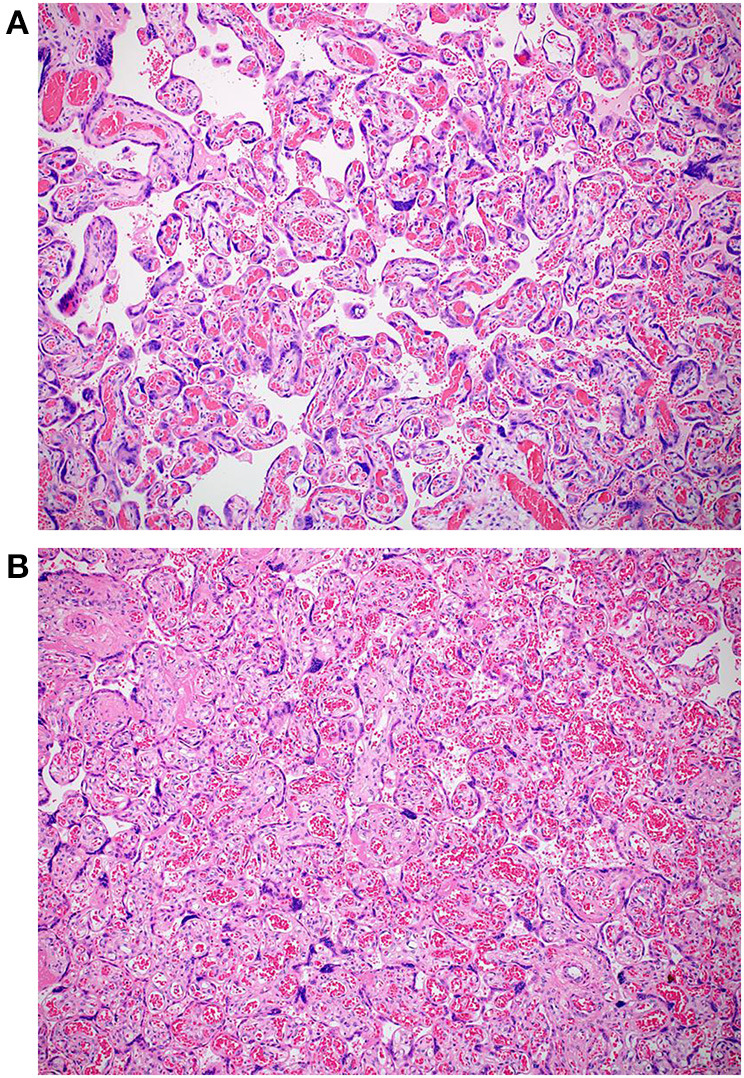
**(A,B)** Microscopic images depicting areas of chorangiosis in case 1 (Hematoxylin and eosin stain, magnification x100).

The macroscopic examination of the placenta showed a singleton placental disc with complete cotyledons and a normal vascular distribution. The fetal membranes were translucent. The umbilical cord was trivascular but hypocoiled (<1 complete spiral for ~5 cm of cord) (Figure 3). Microscopically, the chorionic villi were compatible with the given gestational age with few intervillous hematomas (Figures 4A,B) and scattered areas of chorangiosis (Figures 5A,B). There were no features of maternal vascular malperfusion or inflammation. The microscopic examination of the fetal membranes and umbilical cord was unremarkable. *In situ* hybridization and immunohistochemical staining for SARS-CoV-2 were unavailable in our institution.

Over the course of 12 days, a total of seven samples for COVID-19 PCR from the infant were tested, all of which returned negative. During hospitalization, no expressed breastmilk nor direct breastfeeding was provided; however, breast feeding was recommended upon discharge, as the mother's COVID-19 PCR swab was negative at 10 days after delivery.

### Case 2

A 32-week (gestational age) female newborn, weighing 1,700 g, was delivered by an emergency cesarean section, due to placental abruption, to an un-booked 30-year-old, primigravida mother, at KSUMC in Riyadh, KSA. At the time of admission, a nasopharyngeal swab for SARS-CoV-2 was obtained from the mother, and immediately after birth, the baby was separated from her, in compliance with the hospital policy and due to her mild respiratory symptoms.

**Figure 6:**
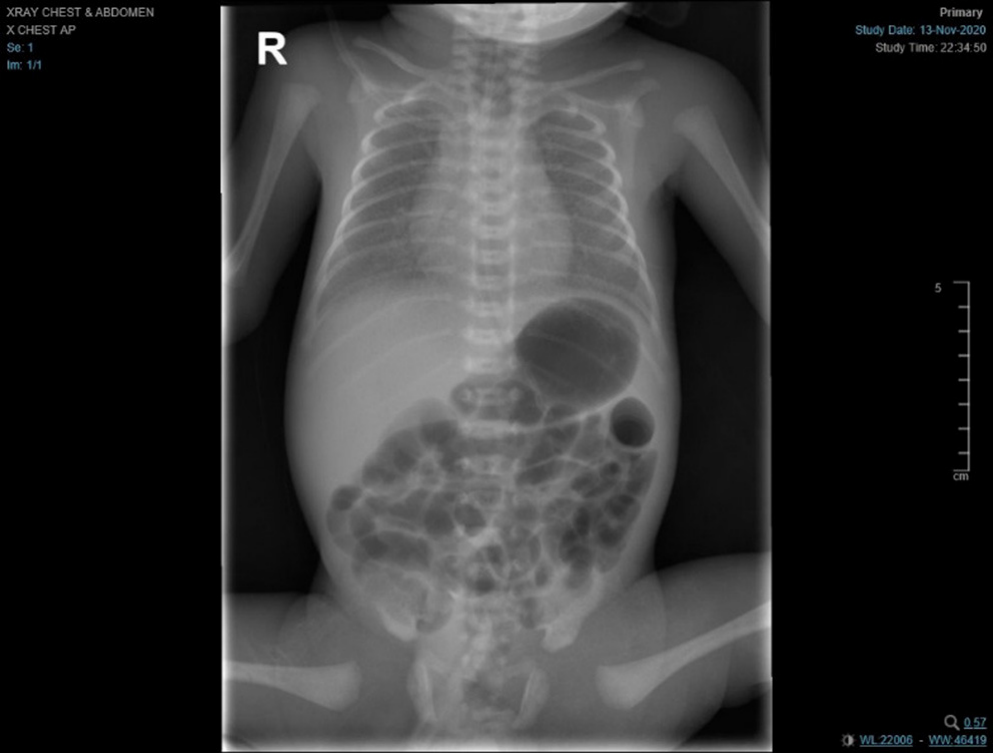
Chest radiograph showed bilateral ground glass appearance with bilateral haziness and good lung volume.

At delivery, the baby required positive pressure ventilation (PPV) for a total of 2 min, because of grunting, and then received nasal CPAP. Apgar scores were 7 and 8 at 1 and 5 min, respectively. The baby was transferred in a closed incubator to an isolation room, as her mother's COVID-19 PCR swab result was still pending. Shortly after birth, the baby developed respiratory distress with an increased oxygen requirement (FiO_2_ of 35%) and required non-invasive mechanical ventilation (NIMV). A limited septic work-up was performed; however, the baby received ampicillin and gentamicin for a total of 10 days despite negative cultures due to elevation in inflammatory markers (Procalcitonin = 0.19 ng/mL). Ten h after delivery, the mother's swab result turned out to be positive for SARS-CoV-2. The baby then was screened by (RT-PCR), which returned positive at 24 h of life. The chest radiograph showed bilateral ground glass appearance with bilateral haziness and good lung volume (see Figure 6), so she was managed as a case of respiratory distress syndrome (RDS) with possible COVID-19 infection.

The initial CBC on the 1st day of life was within normal limits (Table 2), but on the 3rd day of life the blood work revealed leukocytopenia, lymphocytopenia, and normal Hb and platelet counts. The coagulation profile was deranged in the context of elevated liver enzymes with high D-dimer. The inflammatory markers (serum ferritin and procalcitonin) levels were elevated, and serum albumin concentration was low. Furthermore, elevated troponin and markedly elevated BNP suggested the presence of myocarditis (Table 2). On the 4th day of life, an echocardiograph showed normal cardiac anatomy and function. The diagnosis of multi-system inflammatory syndrome in children (MIS-C) was made, so two doses of IVIG (1 g/kg/day) and intravenous hydrocortisone (0.5 mg/kg every 12 h for 7 days) were administered. Hydrocortisone treatment was discontinued without tapering as the infant developed easy bruising.

**Table 2:** Laboratory characteristics of case 2.

**General hematology**	**DOL: 1**	**DOL: 2**	**DOL: 3**	**DOL: 4**	**DOL: 5**	**DOL: 7**	**DOL: 9**	**DOL: 10**	**DOL: 12**	**DOL: 16**	**DOL: 25**
WBC	7.600 (L)		8.700		3.4	2.5	9.3	8.2			
Hgb	174		175		177	107 (L)	144	139 (L)			
Hct	52.5		52		52.6	40.7 (L)	43.3	43.1 (L)			
Platelets	234		187		158	126 (L)	190	271			
Neutro Auto#	2.6 (L)		5 (L)		1.2 (L)	1.3 (L)	2.6	3.1			
Neutro Auto %	34.5 (L)		57.4		35.7 (L)	51 (L)	28.4 (L)	38.1			
Lympho Auto#	4		2.7		1.4 (L)	1.1 (L)	4.2	2.8			
Lympho Auto %	53 (H)		31.6 (H)		41.6 (H)	43 (H)	44.9	33.8 (L)			
ESR					2			2			
PT					19.3 (H)	14.9					
INR					1.43 (H)	1.09					
APTT					54.4 (H)	35.5					
Fibrinogin					2.21						
D-Dimer					1.06 (H)						
ALT		11 (L)		10 (L)		10 (L)		11 (L)			
AST		80 (H)		40 (H)		39 (H)		40 (H)			
BUN		3.2	3.4	2.6			2.6	3.7			
Creatinine Lvl		41	54	49			41 (H)	42 (H)			
LDH				718 (H)				515 (H)			
GGT		141 (H)		118 (H)		108 (H)		86 (H)			
Ferrtin					567.1 (H)			429.1 (H)			
CRP				0.839				2.02			
BNP				5,610 (H)					5,636 (H)		2,223.0 (H)
Total CK				167				46			
Troponin-I					51.9 (H)				28.8 (H)	100.6	
SARS-COV-2 IgG									0.03		
SARS-COV-2 IgG Interp									Negative		
COVID-19		Positive	Positive		Positive			Positive			

On the 5th day of life, the repeated blood work showed further drop in the WBC, lymphocytes, and platelet counts and the hemoglobin concentration as well, which led to her repeated PRBCs transfusions (Table 2). On the 10th day of life, respiratory support was changed to nasal cannula, and 3 days later, the infant was able to maintain SpO_2_ within normal limits without any support. On the 11th day of life the laboratory investigations showed improvement in the serum ferritin and LDH levels, normalization of the CBC and liver enzymes. Furthermore, troponin level was reduced almost by 50%, but the BNP level remained high (Table 2).

During hospitalization, a total of six swabs for PCR were done, the first five swabs were positive; however, COVID-19 IgM and IgG were non-reactive (Table 2). The last swab for PCR was done on day 29 of life and was negative.

**Figure 7:**
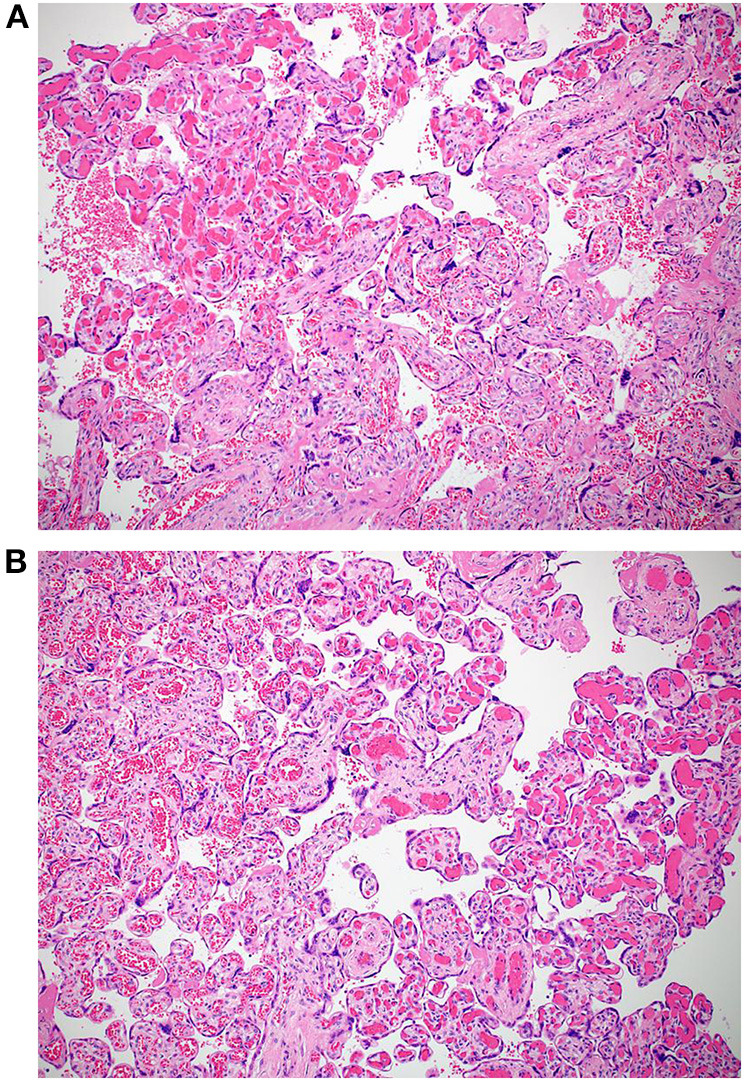
**(A,B)** Photomicrographs showing areas of chorangiosis in case 2 (Hematoxylin and eosin stain, magnification x100).

The macroscopic examination of the placenta showed a singleton placental disc with complete cotyledons and a normal vascular distribution. The fetal membranes were translucent. The umbilical cord was trivascular but short (around 17 cm). Microscopically, the chorionic villi showed areas of delayed villous maturation and focal chorangiosis (Figures 7A,B). There were no features of maternal vascular malperfusion or inflammation. The microscopic examination of the fetal membranes and umbilical cord was unremarkable. *In situ* hybridization and immunohistochemical staining for SARS-CoV-2 were unavailable in our institution.

On the 3rd day post-delivery, the mother was free of symptoms and discharged home in good condition. At the age of 30 days, the infant's weight reached 2,000 g and she was discharged home, too. Notably, she was not offered her own mother's milk throughout hospitalization.

## Discussion

In this report we have accounted for two cases of COVID-19 disease during pregnancy, contributing to premature labor, respiratory distress, and multisystem organ involvement. This adds to the growing evidence pointing to the potentially devastating maternal and neonatal outcomes (9, 10). The most frequently encountered presentation of the disease during the neonatal period is asymptomatic or mild infection and the seemingly uncomplicated course of most cases (2, 11, 12). The literature, however, indicates that a few cases need intensive care and fewer cases required invasive ventilation with extracorporeal membrane oxygenation (ECMO) (3, 13). Current evidence is not clear whether pregnancy-related immune modulation alters the path of the disease by suppressing the exaggerated inflammatory responses observed in this disease and correlated with a bad prognosis (4, 14). It is important to keep in mind that in our study both mothers and one infant had positive nasopharyngeal swabs for SARS-CoV-2, whereas the other infant had high specific SARS-CoV-2 immunoglobulin (IgG) within 7 days of life, despite her negative swabs for viral detection. The sensitivity and specificity stated by the manufacturer for IgM detection were 88 and 99%, respectively, and for IgG were 98 and 98% (11). The sensitivity of RT-PCR test on nasal swabs obtained from adult patients is 63% (12). Although the nasopharyngeal swab for SARS-CoV-2 was negative and IgM levels were not obtained in the first patient, tests for specific SARS-CoV2 immunoglobulins were positive.

This may reflect a more complicated intrauterine transmission process that goes back to the first maternal COVID-19 infection in the second trimester, which could explain also the negative result of the nasopharyngeal swab at birth.

An intrauterine infection is suggested when a mother tests positive for SARS-CoV-2 within the period of 14 days before birth to 2 days after birth, with the detection of the virus in the neonatal respiratory tract in the first 24 h of life, with either the persistence of swab positivity after 24 h of postnatal life or positive SARS-CoV-2 IgM in the first 7 days of life (15). In one report, the vertical transmission was presumed in a newborn by high specific SARS-CoV-2 immunoglobulins level obtained at 2 h of age, but all 5 RT-PCR tests on nasopharyngeal swabs were negative (16). Zeng et al. included six mothers with reported COVID-19 disease, SARS-CoV-2 was not found in the serum or throat swab by RT-PCR in any of their newborns, with elevated IgG concentrations in five babies, but IgM was detected in two infants (11). The possibility of vertical transmission in these cases is uncertain. Of note, in the second case, the mother was infected with SARS-CoV-2 in a period of 14 days before birth to more than 2 days after birth, with a positive nasopharyngeal swab for SARS-CoV-2 in the first 24 h of life of the infant, with persistently positive swabs for 15 days of postnatal life, favoring the conclusion of intrauterine transmission of SARS-CoV-2, despite the negative IgM in the first few days. In these two infants, we assume that the risk of infection during cesarean section or in the postnatal period was extremely low, due to the strict isolation steps applied immediately after delivery, which favors the suspicion of SARS-CoV-2 *in utero* transmission. Previous reports have not indicated maternal-fetal transmission of SARS-CoV-2, including negative checks of amniotic fluid, umbilical cord blood, vaginal swabs, and breast milk (2, 4, 5, 17, 18).

The histomorphological features described in placentas from SARS-CoV-2-positive women are still limited to case reports or case series with no pathognomonic findings as reported by Sharps et al. (19). Wong et al. also recently reviewed 17 studies demonstrating evidence of SARS-CoV-2 in third and second-trimester placenta cells/tissues (20). The most common finding was changes related to maternal vascular malperfusion (37.8%), including villous infarction, increased perivillous/intervillous fibrin, and decidual vasculopathy. The second most common finding was inflammation (34.7%), including features of maternal inflammatory response (subchorionitis/chorionitis and chorioamnionitis) or fetal inflammatory response (chorionic or fetal vasculitis). Fetal vascular malperfusion (FVM) accounted for 9.2% of all findings, including most frequently chorangiosis (33.3%) and villous stromal-vascular karyorrhexis (33.3%), followed by delayed villous maturation, avascular villi, and fetal vascular thrombi. In their review, 2% of all placentas did not show any pathological findings. Our findings are not inconsistent with the published data. Although none of the cases showed features of maternal malperfusion, both showed foci of chorangiosis consistent with fetal vascular malperfusion. One case showed a few intervillous hematomas, and the other showed delayed villous maturation, also a feature of FVM. Both cases also had umbilical cord abnormalities (a reduced coiling index in the first case and a short cord in the second). These findings are also consistent with a few published reports (21, 22). Chronic histiocytic intervillositis is one of the reported findings in placentas from SARS-CoV-2-positive women. Two cases of chronic histiocytic intervillositis have been described in each of the three published case series (23–25). However, this finding was not observed in any of our two cases.

Unfortunately, immunolocalization of the viral mRNA by *in situ* hybridization (ISH) and immunohistochemistry (IHC) for SARS-CoV-2 are unavailable in our institution. Eight studies included in the review by Wong et al. did not perform ISH, IHC, or reverse transcription-polymerase chain reaction (RT-PCR). Among the studies that performed any of the above-mentioned ancillary techniques, two showed negative RT-PCR on the examined placentas, and two showed negative ISH and IHC results (20).

In view of the non-specific and somewhat subjective histological features of placental vascular malperfusion, further research is warranted, including a standardized examination of placentas from SARS-CoV-2-positive women and matched negative controls by specialized pathologists blind to the patient's SARS-CoV-2 status.

Fetal inflammatory response syndrome (FIRS) is the multisystem inflammatory syndrome in fetuses. Neonates impacted by FIRS would present with varying degrees of multi-organ system involvement and high morbidity (26, 27). FIRS is characterized by elevated concentrations of IL-6, which correlate sometimes with leukocytosis and neutrophilia; it is often associated with enhanced fetal plasma concentrations of tumor necrosis factor receptors and CRP, too (17). This was demonstrated in the first case with the evidence of inflammation and multisystem involvement vs. multi system inflammatory system in the neonate. Furthermore, placental pathology entails chorionic vasculitis in certain pregnancies (22). Recurrent infarctions seen on placental pathology are associated with vascular injury and may be due to inflammation secondary to maternal viral infection. These results, in addition to late-onset fever and multi-organ involvement, with no definite microbial source, are more likely to suggest FIRS in the first case. FIRS due to maternal SARS-CoV-2 infection can contribute to severe neonatal morbidity (7). The management of patients with FIRS requires shock control, immunomodulatory therapy, and the usage of thrombo-prophylaxis agents.

Due to mothers' declining further testing, we performed histopathological examination of the placenta but were unable to investigate the presence of the virus in the amniotic fluid, placental tissue, or cord blood samples. Performing these studies would further clarify the pathogenesis of this illness and augment our conclusions of vertical transmission of SARS-CoV-2 virus. This report suggests the presence of multi-system inflammatory syndrome in the neonates (MIS-N) along with MIS-C, which is an established entity of COVID-19 disease among children. The second case we present supports the possibility of vertical transmission of SARS-CoV-2 with secondary multi-system involvement and inflammation. In this case SARS-CoV-2 PCR was persistently positive with liver and cardiac involvement. Having no fever throughout the course of illness suggests that neonates respond to infection with SARS-CoV-2 differently compared with children. It might be important to reevaluate the current criteria of MIS-C to be generalizable to neonates or to develop new criteria for diagnosis of multi-system inflammatory syndrome in neonates.

## Data Availability Statement

The original contributions presented in the study are included in the article/Supplementary Materials, further inquiries can be directed to the corresponding author/s.

## Ethics Statement

The studies involving human participants were reviewed and approved by King Saud University E-21-5927. Written informed consent to participate in this study was provided by the participants' legal guardian/next of kin. Written informed consent was obtained from the individual(s), and minor(s)' legal guardian/next of kin, for the publication of any potentially identifiable images or data included in this article.

## Author Contributions

LS and FA conceptualized and designed the study, drafted the initial manuscript, and reviewed and revised the manuscript. KA, HB, and AH designed the data collection instruments, collected data, carried out the initial analyses, and reviewed and revised the manuscript. AJ and AMA conceptualized and designed the study, coordinated and supervised data collection, and critically reviewed the manuscript for important intellectual content. All authors approved the final manuscript as submitted and agree to be accountable for all aspects of the work.

## Conflict of Interest

The authors declare that the research was conducted in the absence of any commercial or financial relationships that could be construed as a potential conflict of interest.
